# Finding Consensus About the Level of Medication Safety in a Hospital Setting: Development and an Example of Application of a Modified Delphi Method

**DOI:** 10.3389/fpubh.2021.630398

**Published:** 2021-09-14

**Authors:** Birgit Böhmdorfer-McNair, Wolfgang Huf, Reinhard Strametz, Michael Nebosis, Florian Pichler, Susanne Melitta Janowitz, Brigitte Ettl

**Affiliations:** ^1^Pharmacy Department, Clinic Hietzing, Vienna Healthcare Group, Vienna, Austria; ^2^Karl Landsteiner Institute for Clinical Risk Management, Vienna, Austria; ^3^Department of Laboratory Medicine, Clinic Hietzing, Vienna Healthcare Group, Vienna, Austria; ^4^Wiesbaden Business School, RheinMain University of Applied Sciences, Wiesbaden, Germany; ^5^Emergency Department, Clinic Hietzing, Vienna Healthcare Group, Vienna, Austria; ^6^Pharmacy Department, University Hospital Vienna, Vienna Healthcare Group, Vienna, Austria; ^7^Medical Directorate, Clinic Hietzing, Vienna Healthcare Group, Vienna, Austria

**Keywords:** medication safety, method development, decision maker, questionnaires, Delphi method, expert elicitation, methodology, consensus

## Abstract

A version of the Institute for Safe Medication Practices (ISMP) questionnaire adapted to the Austrian inpatient setting was used to sample the estimates of a group of experts regarding the level of medication safety in a level II hospital. To synthesize expert opinions on a group level reproducibly, classical Delphi method elements were combined with an item weight and performance weight decision-maker. This newly developed information synthesis method was applied to the sample dataset to examine method applicability. Method descriptions and flow diagrams were generated. Applicability was then tested by creating a synthesis of individual questionnaires. An estimate of the level of medication safety in an Austrian level II hospital was, thus, generated. Over the past two decades, initiatives regarding patient safety, in general, and medication safety, in particular, have been gaining momentum. Questionnaires are state of the art for assessing medication practice in healthcare facilities. Acquiring consistent data about medication in the complex setting of a hospital, however, has not been standardized. There are no publicly available benchmark datasets and, in particular, there is no published method to reliably synthesize expertise regarding medication safety on an expert group level. The group-level information synthesis method developed in this study has the potential to synthesize information about the level of medication safety in a hospital setting more reliably than unstructured approaches. A medication safety level estimate for a representative Austrian level II hospital was generated. Further studies are needed to establish convergence characteristics and benchmarks for medication safety on a larger scale.

## Introduction

Over the past two decades, patient safety movements, in general, and the medication safety movement, in particular, have gained momentum (1, 2). However, the ideal path to improvement for either of them is far from clear. Nevertheless, assessing reality in the form of current practice is the first step toward changing for the better (3, 4). Getting consistent data about medication in the complex setting of a hospital, however, is tricky and time-consuming since multiple aspects of the issues are embedded in different settings and professions. For example, the simple question of the responsibility for maintenance and checking of the temperature of refrigerators where drugs are kept in the wards is a matter of shared responsibility between nurses and the technical department, while checking the documentation thereof can be part of the ward inspection done by pharmacists. Refrigerators for blood, for drugs, for studies, or for narcotics that need refrigerating will be dealt with and checked differently.

Questionnaires can be used to assess medication practice in healthcare facilities. To be able to answer questionnaires reliably, however, a thorough and detailed knowledge of the clinical setting is key, and this knowledge can usually not be allocated to a single person (5). Thus, arises the need to elicit and synthesize knowledge from a group of healthcare professionals (6). Currently, there has been no method published for finding consensus regarding the level of medication safety in the complex setting of a hospital.

The central assumption of the approach proposed in this study is that it is not necessary for every person in a clinical setting to answer a questionnaire to get relevant, detailed, and valid data about their work surroundings. The well-considered collection of a group of people in combination with a structured analysis of their answers will be sufficient to accumulate the information required (5, 6). For the specific challenge of estimating the level of medication safety, the composition of such an expert group has been described in 2011 for the *ISMP Medication Safety Self-Assessment for Hospitals* questionnaire (7).

The ISMP questionnaire has been translated and adapted to the Austrian inpatient setting in a project supported by the Austrian Ministry of Health and has since been applied to a group of hospitals in Austria under the title of Austrian Medication Safety Strategy (AMEDISS) (8). However, a structured way for synthesizing information from the described group of experts has neither for the AMEDISS questionnaire nor for the ISMP questionnaire been published.

For disseminated knowledge, purely quantitative methods of synthesis do not appear to be the ideal approach. Hence our choice to approach decision making with a combination of elements from quantitative as well as qualitative research. The purpose of this study is to identify such an approach to eliciting knowledge regarding the level of medication safety in complex clinical settings and to test it on a single hospital dataset.

## Materials and Methods

### Questionnaire

The AMEDISS questionnaire is based on the Institute for Safe Medication Practice assessment for hospitals (7) that was adapted to the Austrian hospital environment (8). It consists of 268 standardized questions, allocated to 10 key elements which have an influence on safe medication use, for example, “communication of drug orders and other drug information.” The AMEDISS questionnaire that was used incorporates the ISMP questionnaire of 2011. A German version is available through the Plattform Patientensicherheit—Amediss (8).

There are five possible answers to each of the 268 questions, ranging from “no activity to implement” through “considered, but not implemented” to “fully implemented throughout.” For this study, “unable to answer” was added as a potential answer to be able to differentiate between whether the experts simply felt they were not familiar with the topic or they omitted to answer the item.

The general principle of every item is: if the grade of implementation of an item is high, this is considered good for medication safety; items that score with low implementation indicate areas for future improvement for the hospital.

### Environment

We collected our data in a public hospital in Vienna, Austria with more than 800 beds. The departments include internal wards, namely, oncology, surgical wards, neurology, and ICUs. The hospital takes part in research and training in several professions and on several professional levels and has a pharmacy on-site. The hospital is part of a large healthcare group.

### Initiation of the Project—Recruiting of the Experts and Monitors

The interdisciplinary team of experts (expert panel) was assembled following the suggestions of the 2011 ISMP Self-Assessment for Hospitals Handbook (7). It consisted of 14 persons, mainly employees of the hospital, women, and men, representing different professions, areas of expertise, and levels of seniority within the hospital: four nurses, six physicians, one midwife, three pharmacists, one IT specialist, and one risk management and quality improvement professional. (The total is larger than 14 due to multiple qualifications.) All members have several excellent qualifications, but the following qualifications were found to be especially valuable in the context of this project: experience in different working areas (such as ICU, surgical and internal wards) and with project participation and project management, experience with different prescribing systems (electronic and paper), and experience with risk management projects.

All the 14 members of the expert panel were asked to answer the AMEDISS questionnaire independently to avoid bias through interaction with other panel members. A researcher-administered survey questionnaire approach was used by one member of the monitoring team who was specifically assigned to that task. One member of the expert team, however, chose a self-administered approach. Answers to the 268 items of the questionnaire were collected, in scoring keys predefined by the questionnaire. Additional comments were either audio-recorded and then transcribed verbatim or put down in writing by the interviewees themselves. This was done according to predefined instructions from the monitor panel, which specified that comments during the interview of a purely conversational character like “could you repeat that question” or “let me think” were excluded from verbatim transcription.

The ISMP Medication Safety Self-Assessment questionnaire for hospitals, which is the basis for the AMEDISS questionnaire, comes with a handbook. This handbook includes general instructions for conducting the self-assessment, such as suggestions for the qualitative and quantitative composition of an interdisciplinary team answering the items.

In contrast to the composition of the expert team, however, the generation of representative answers to the questions is not described. It appears vital for overall answer validity that disseminated knowledge is synthesized in a valid and reproducible manner.

The team conducting the processing and analysis of the data (monitor group) consisted of five persons who work for the hospital, representing different professions, areas of expertise, and levels of seniority within the hospital: two physicians and three pharmacists. In the context of this project, it was found especially helpful that besides their professional core competencies, the members of the monitor team share a broad experience of several years working in the hospital, with management, catastrophe management, quality management, scientific research, every possible aspect of drug management within the hospital context, and IT management among their areas of experience.

Decisions made by the main researchers were discussed in an open and lively fashion, using the principles of a focus group (9).

### Data Analysis

A systematic approach to collecting and assessing data about medication safety in a thorough, systematic, and reproducible way was developed using a subvariant of a classical Delphi model (5) in combination with the elements of item and performance weight decision making (10, 11).

Once the questionnaires were completed by the experts, the data were transferred into spreadsheets, and each item was analyzed individually for statistical interpretability and plausibility by the monitor group, i.e., the team conducting the processing and analysis of the data. Verification of the entries on spreadsheets was done by the monitor team while analyzing and discussing the data was completed on-site by all members of the monitor group. Whenever possible, the answers were cross-checked to establish plausibility, for example, in an ICU-related item, the answers provided by interviewees with expertise in intensive care were compared and checked for plausible conformity. In case of doubt, the monitor team consulted additional experts to provide further information. Further discussion and analysis were then logged on spreadsheets, using predefined number codes.

For the analysis of additional verbal or written comments, systematic text condensation was planned as the method of choice (12) to gather potential additional insights on medication safety issues, but since there were only very few of these comments, no systematic text condensation was conducted.

The presentation of the findings takes into consideration the requirements of the Standards for Reporting Qualitative Research (SRQR) (13).

It took about 5 h of meeting time to inform experts about the background, start, and milestones of the project. The average time of one interview with one expert was about 3 h including the compilation of numerical data in spreadsheets. The monitor team met over about a year for meetings with a total length of 25 h to discuss several aspects of the project. Extra time was needed for project management, method development, and literature research.

## Results

### Expert-Level Results

All 14 interviews were completed as planned, and additional inquiries for certain items were conducted as suggested by the 2011 ISMP Self-Assessment for Hospitals Handbook (7). These answers of single experts to certain items were asked for and collected by email and phone.

Once the questionnaires were answered by the interdisciplinary team and returned to the team conducting the processing and analysis of the data (“monitor group”), it was seen that a clear answer could be generated by just combining the majority of the 14 answers of the 14 experts for only 35% of the items. The interpretation, combination, and aggregation of the answers for the remaining items turned out to be worthy of discussion. These and the consequent choice of appropriate decision-maker are described in the figures and the following paragraph.

### Synthesis Methodology

The method developed is described in four detailed graphs (refer graphs “Task_1: Initiation the project and getting individual answers to the questions,” “Task_2: Cross-reference: Compilation of questionnaires,” “Task_3: Evaluation of single items of the questionnaire in synoptic presentation,” and “Task_4: Consequences of an invalid result”).

The graphs describing the tasks show every step of the way, thus making it possible for everybody to retrace and reproduce the method in their setting.

**Figure 1:**
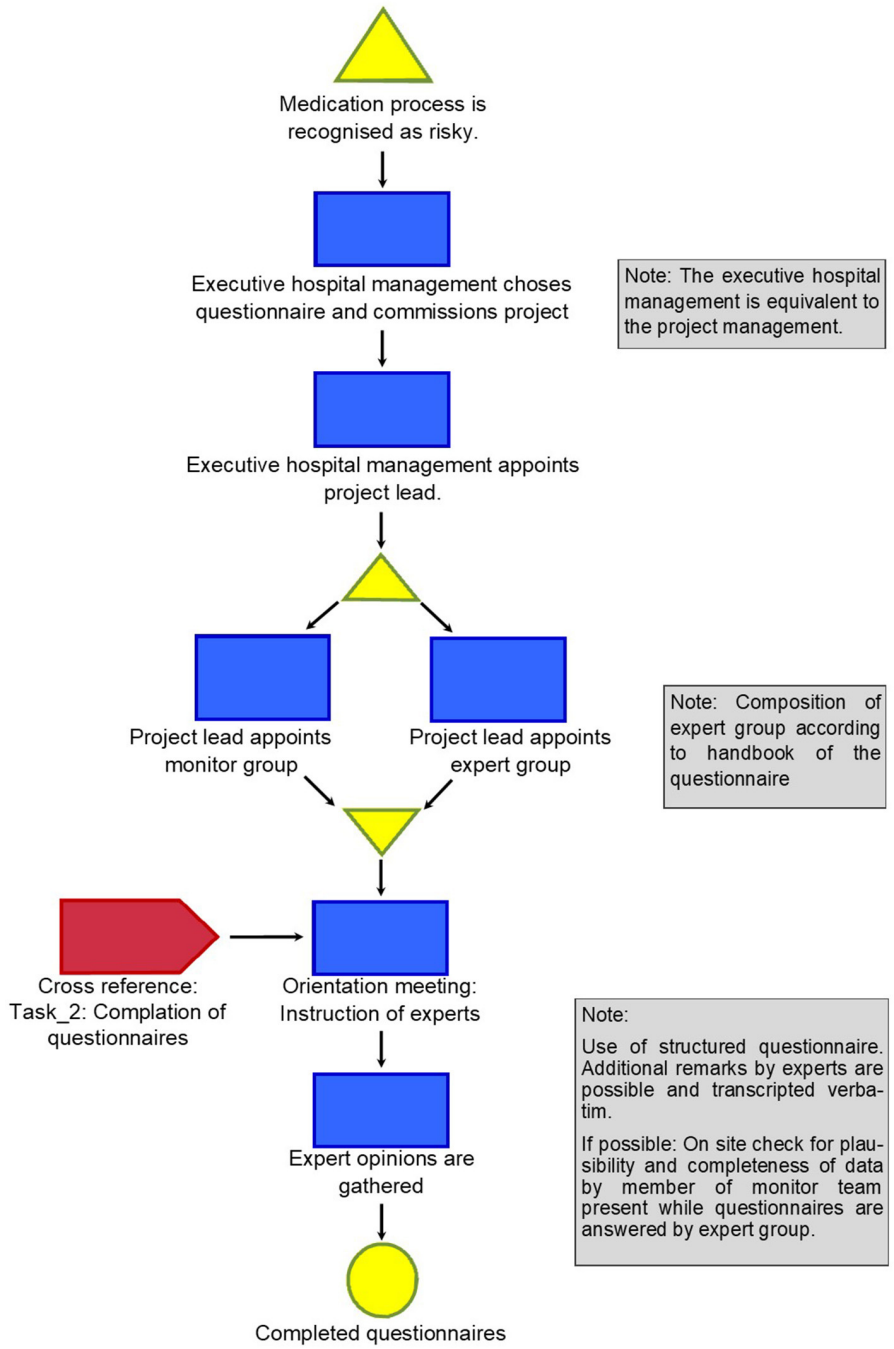
Task_1: Initiating the project and getting individual answers to the questionnaire.

Project initiation and gathering of expert opinions are visualized in Figure 1. Expert opinions are gathered using a questionnaire with a survey and data collection. This is done in the style of the first round of a classical Delphi method using a group of experts that do not interact among themselves to avoid cross-interference and by offering a choice of predetermined answers to each item of the questionnaire.

**Figure 2:**
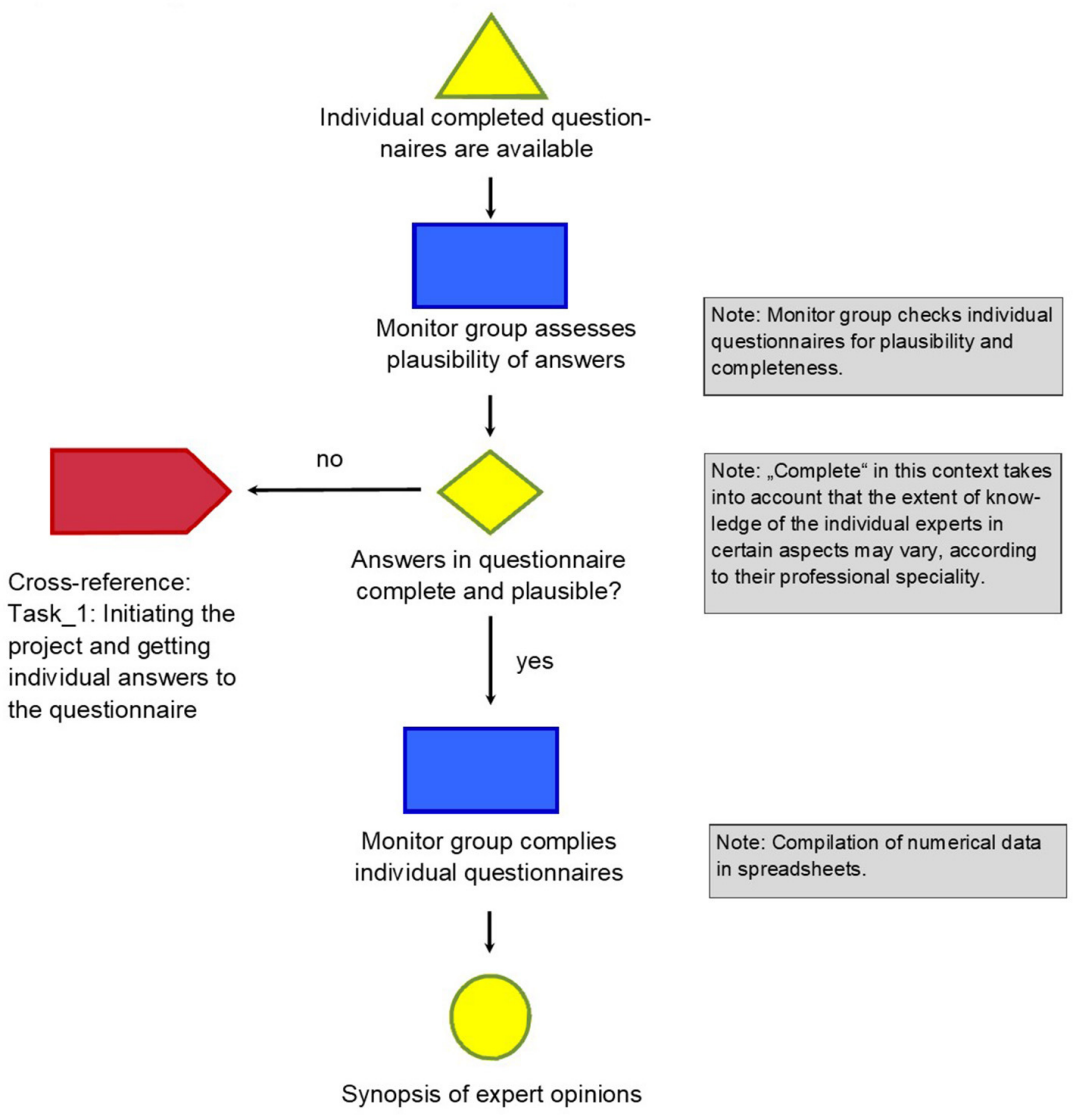
Task_2: Cross-reference: Compilation of questionnaires.

Figure 2 describes how the individual questionnaires are compiled to create a synopsis of expert opinions, checked for plausibility and completeness, and categorized with an ordinal scale. Optional additional statements of the interviewees are collected verbatim to allow further structured interpretation if so desired, for example, by systematic text condensation (11). So far, this approach follows a classical Delphi approach to gathering data in a complex matter when precise answers are tricky to impossible to obtain from a single individual. This Delphi approach attributes equal weight to each answer from every single expert, a principle called equal weight decision-maker.

**Figure 3:**
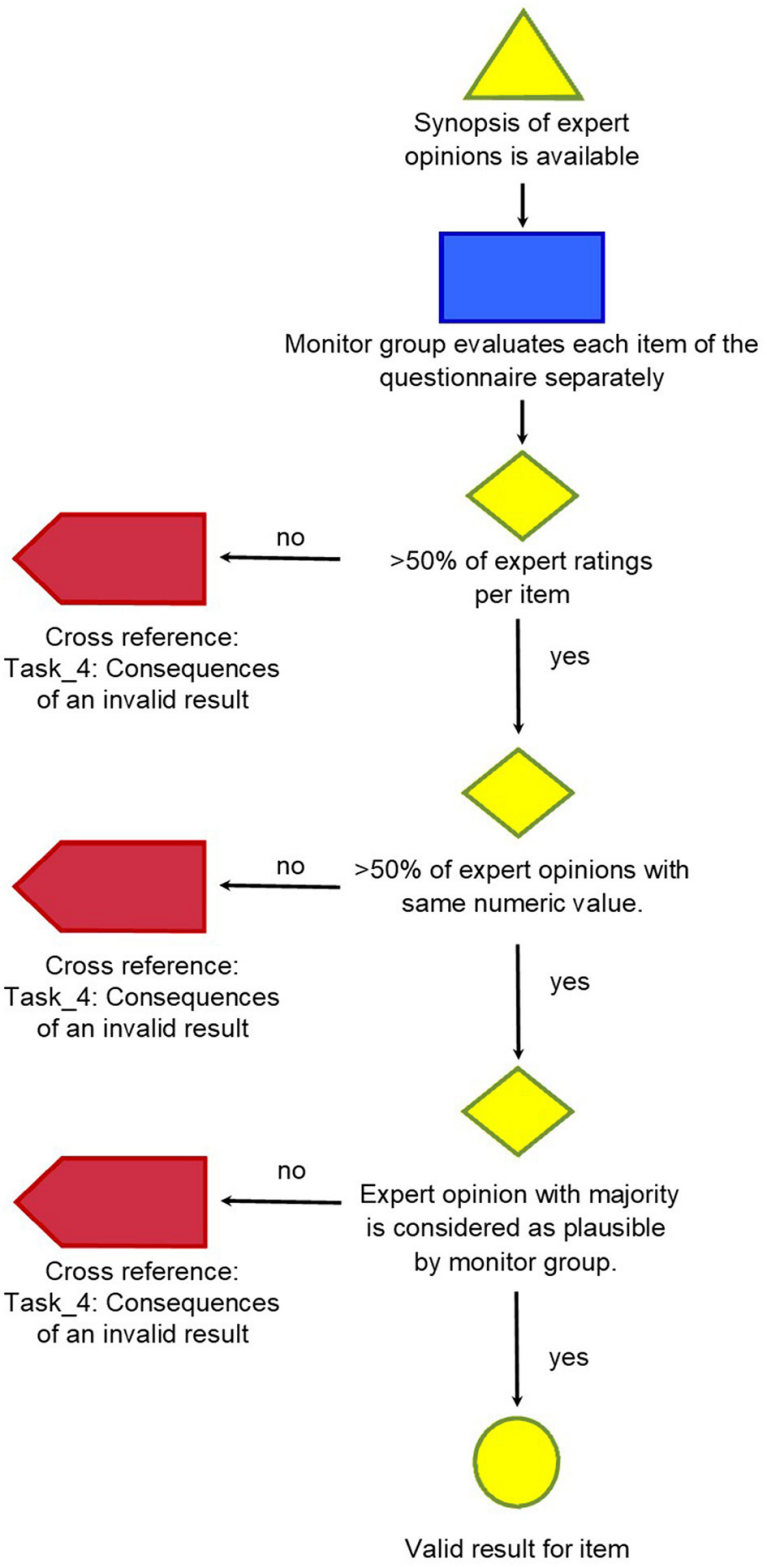
Task_3: Evaluation of single items of the questionnaire in synoptic presentation.

It was found that satisfactory results—which were defined as more than 50% of expert ratings per item, more than 50% expert opinions with the same numeric value and rating considered as plausible by the monitor group—were reached for 35% of the items of the questionnaire with this first round of Delphi. This is described in Figure 3.

The classical Delphi approach would then reiterate the items that were not answered to satisfaction with the same set of experts as in the first round, additionally providing them with statistical data about the collective answer patterns in the previous round. We decided not to do that. Rather than start a second round of Delphi with the items which yielded invalid results so far, the possible causes were analyzed to search for a further approach for these remaining items.

Four reasons were identified, that necessitated variation of the classical Delphi method for synthesizing expert opinions:

1. Reiteration with a classical Delphi approach is usually only required for a minority of the items of the first round. In this study, this would have meant reiterating more than half of the questions. This was interpreted as a definite clue, that persevering with a classical Delphi approach was not ideally suited for the task ahead.
2. Delphi is generally used to create predictions, i.e., speculations about future events or assessment probabilities. We postulated that when it comes to matters of facts about current medication practice, this knowledge is not hypothetical guesswork of probabilities, but existent, albeit not necessarily known to everybody. Therefore, the reiteration of a question will not be likely to yield any hidden knowledge, if the same question were to be put to the same set of experts, whose professional experience, training, and knowledge would most likely not have undergone massive changes in the next round of Delphi, which is usually done within days or weeks.
3. In addition, there is a particular feature of questionnaires assessing medication practice, like the ISMP questionnaire, to be considered: in these questionnaires, is of interest that the importance of each topic, or the level of implementation of each aspect, varies across the whole healthcare facility. Special on-site training standards might, for example, be implemented only in special professional groups, or electronic drug prescribing might be limited to ICUs. To reflect these aspects, the answers to the questionnaires will differ accordingly, and the strategic analysis of the monitor group will be necessary to translate and summarize these single viewpoints according to the level of implementation. This will then lead to plausible results for several questionnaire items that yielded inconsistent results when analyzed strictly by the mean or median response, but that makes perfect sense, once one considers the pattern of different levels of implementation.

**Figure 4:**
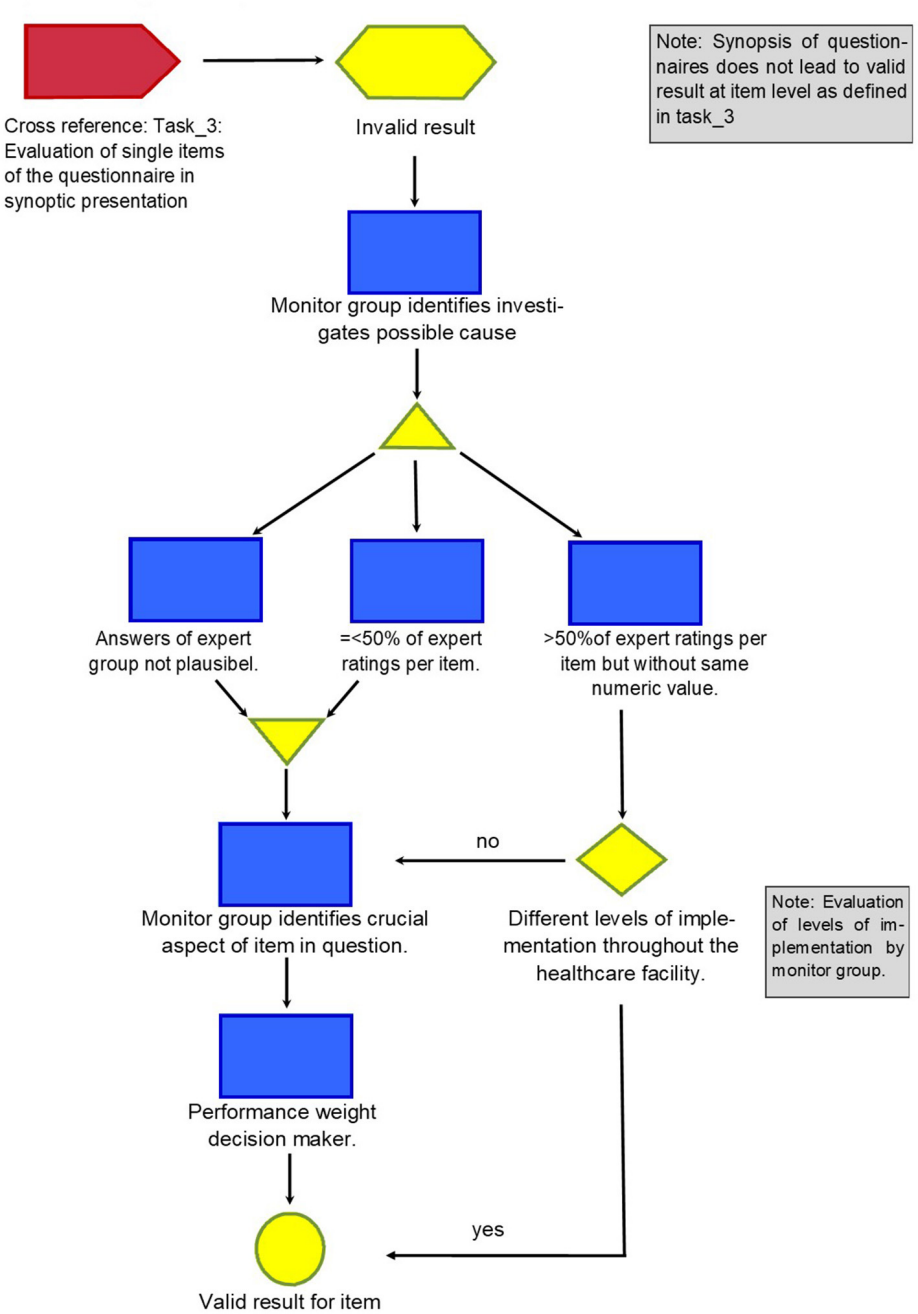
Task_4: Consequences of an invalid result.

From the perspective of monitors, the crucial point is, therefore, to either realize that a heterogeneous answer pattern coincides with a heterogeneous pattern of implementation of medical practice within the hospital or to identify the specific expert who is best informed about a particular aspect that is not generally shared by the experts in the first round [6]. This process is described in Figure 4.

The monitor group found that different levels of implementation were the answer to 26% of the remaining questions, but that still left 37% of the items of the questionnaire without satisfactory answers. For these, the monitor group identified potential experts as so-called “performance weight decision makers”: When different weights are attributed to different experts, this is called performance weight decision-makers (10, 11). Doing this, the weight of the opinion of experts varies for each item, thus resulting in a method called “item-based decision maker.” This gives credit to the fact that in complex matters, like drug therapy safety, not every expert can have the same level of expertise and experience for every aspect. Ideally, the performance weight decision-making experts were already part of the initial expert group.

When using performance weight decision-makers, Cooke (10) suggests calibrating the assessment of experts through calibration questions. This was not considered necessary in this setting, since the required qualifications could be deduced from the qualification and work surroundings of the persons in question, who were well known to the monitor team, and therefore, the answers required were not unknown probabilities but facts. Already known and established levels of qualification and expertise were, therefore, not challenged.

For example, for questions about cytotoxic drug preparation, the hospital pharmacist specializing in cytotoxic drug preparation, who was part of the expert panel, would be identified as an item-based decision-maker when necessary, by the monitor group. When not identifying a potential item-based decision-maker for a specific item of the questionnaire within the expert group, the monitor group would then look, secondarily, among the monitor group, and in some cases, additional experts were consulted.

The experts for the item weight decision-making were identified by the monitor group according to the expertise requested by the respective item. More than half (55%) of the items were answered by assigning the weight of the decision of one or more members of the initial expert panel. About a third (28%) of the items could be answered by members of the monitor group. The remaining items were processed either by recruiting additional experts or combining the answers of two of these three groups. The additional expert knowledge for the fields of nutrition, materials logistics, medical engineering, and for special aspects of anesthesiology and intensive care was required.

This combination of equal weight decision-maker plus considering different levels of implementation plus item weight decision-maker with performance weight when necessary yielded results for the 268 items of the AMEDISS questionnaire as follows.

**Figure 5:**
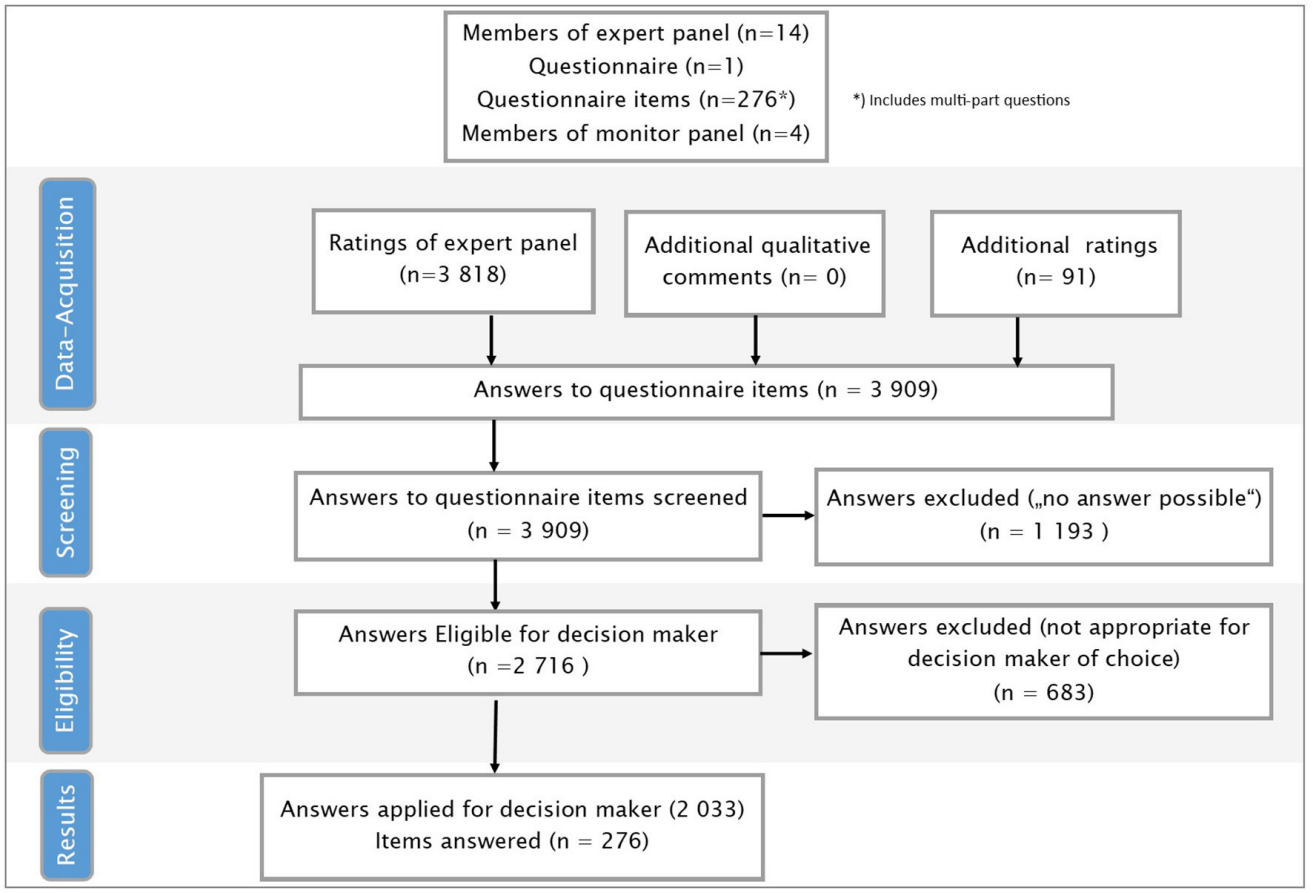
Flowchart decision-maker.

Only 35% of these answers were generated through the first round of a classical Delphi approach using an equal weight decision-maker where the opinion of each expert is attributed equal weight. For 26% of the items, plausible answers resulted from interpreting the answers of experts by taking into account different levels of implementation throughout the healthcare facility. Very few items (3%) of the AMEDISS questionnaire did not apply to our specific setting. For the remaining items, i.e., 36%, a performance weight decision-maker was applied. We describe these results in Figure 5.

### Group-Level Results

Only four questions (i.e., about 1.5%) of the questionnaire out of a total of 268 needed additional revision after implementing the method in this study are described. Causes for revision were items in which different interpretations of the wording caused inhomogeneous answers (3) and one single case of human error, where the scoring of one expert in their respective field of expertise was identified as erroneous by the monitor team and was, therefore, not taken into consideration.

## Discussion

### Links to Empirical Data, Integration With Prior Work, Implications, Transferability, and Contributions

Medication safety is an important topic. Questionnaires like the 2011 ISMP Medication Safety Self-Assessment® for Hospitals (7) are already widely used (14) and will most likely gain more importance to assess and potentially improve medication practice in healthcare facilities. We are aware of publications describing combinations of the Delphi method with other qualitative techniques (15, 16). To our knowledge, no other group describes their experiences and findings using a combination of a Delphi approach, complemented with the principles of item weight decision making and performance weight decision making.

Focus groups (9) are a very good tool in patient safety as well but would instead have given us information about how people feel about the subject of patient safety rather than describe the current status quo. We decided to go for a modified version of Delphi to encourage individual responses to every question from every participant whenever possible.

In the concrete case of this study, consistent data about medication practice in a large Viennese public hospital were assembled. Medication is the most prominent source of unintended and preventable adverse events in the inpatient setting (1) with a substantial clinical and economic burden (3), thus the focus on assessing medication safety was chosen by the hospital management. That data were to be used to assess the present situation and learn how to identify areas of improvement.

Order and method are important in generating trustworthy data, especially in complex systems like a healthcare setting. Using the method presented in this study comes with several advantages:

1. This method can be generalized for use in similar settings and objectives. This will save future investigations time and effort.
2. This method structures the elicitation of data and providing the sound documentation necessary for easy traceability of data.
3. This method uses a subvariant of a classical Delphi model in combination with the element of item weight and performance weight decision making. These methods are recognized as tools with a scientific background.
4. This method is described in detailed graphs, allowing retracing and reproduction.

Accepting the importance of expert judgment in a scientific context is a sound concept (6, 17). There are several areas of expertise, where most people will agree that potential danger (like erroneous drug use leading to bodily harm) is plausibly possible, but the actual critical question is the evaluation, of how likely the scenario is, how severe the damage is going to be, and when it is going to happen. Especially the recent developments in the COVID-pandemic are a showcase demonstration that in areas where lots of data are available, but data interpretation and prognosis are crucial. Expert opinions or combined expert opinions are quite often the resources of choice for major decisions, for example, in governmental health strategies.

Comparing experiences of data acquisition with questionnaires among colleagues, it can be found that the method presented in this article can be considered the “intuitive method of choice” by most people faced with the task of compiling several questionnaires into one answer per item. However, we did not find this method, nor a similar method, in the literature we consulted. Based on the literature available to us, we conclude that the method hereby presented can probably be transferred to similar settings. This conclusion is supported by the guidance provided with the ISMP handbook for the questionnaire (7), which provides comparatively limited instructions, in themselves considered to be sufficient guidance for conducting the survey. Similar issues of healthcare are covered by the same principle of using a questionnaire (18, 19). Using a structured method with a transparent and well-documented procedure adds value and saves time.

Answers to each item of the questionnaire were found by engaging a diversity of different professions and levels of hierarchy within the hospital and encouraging reflection and discussion about medication safety issues throughout the process. Thus, this process itself helps to heighten the risk awareness within the hospital in comparison to a single person filling out the questionnaire on their own (20).

### Limitations and Strengths

Implementation of the approach described in this study crucially depends on the existence of a constructive organizational culture and supportive hospital management. In the case of this study, research was made possible by the explicit encouragement and commitment of hospital management in general and by the medical director and the head of nursing in particular. In a less supportive and professionally diversified environment, systematic analysis on-site may be difficult or even impossible, however, appropriate the method chosen might be in principle.

A limitation in the quality of the answers to the questionnaire lies in the choice of experts and monitors. To limit selection bias, we were, therefore, following the suggestions of the ISPM manual (7) as closely as possible in selecting our experts. The interviews to collect answers to the questionnaire were conducted by a research assistant who was at that time not connected to any of the departments to limit social bias and authority bias, and who stuck to the questionnaire text literally to avoid interviewer bias. We chose interviews with single experts rather than groups, to avoid authority bias, conformity bias, and in-group bias.

The usual tests for validity and reliability that are established in quantitative research, cannot be transferred to qualitative research (21). However, to ensure the credibility of our research, as described in section 1.4, we took the requirements of the SRQR (13) into consideration and the study was conducted in a spirit of reflexivity, using peer debriefing to establish face validity, audio records for the repeated revisiting of data if considered necessary, and maintaining transparent and clear description of the research process from the start to the end. The applicability of our method to other contexts can ultimately only be tested by the application of our method by other study groups, which is why we would like to share our thoughts with the scientific community in this article.

Another limitation is the use of a questionnaire in itself. A questionnaire can only map selected parts of hospital practice and might leave out other areas of potential relevance. Using a questionnaire with predefined items and thereby choosing certain aspects to map a high dimensional space to one or a few dimensions can be helpful, but is also disputable (22).

Finally, the set of assumptions might limit the applicability and generalizability of study results. Important assumptions are, for example, that there indeed exists a potential expert or group of experts for any given item of the questionnaire and that these experts are correctly identified for answering the questionnaire. Most of the assumptions made in this study, however, are basic limitations of any type of Delphi method, or indeed any expert elicitation in general (5, 6, 23, 24).

### Summary of Results

The following results were found:

1. All interviews were completed and produced 14 completed questionnaires.
2. A method of choosing how the decision was generated was created for each item.
3. The 14 questionnaires were combined to one answer per item.
4. The answers give a starting point to describe and potentially further improve aspects of medication safety in a hospital in Vienna, Austria.
5. The method of the decision-making process was documented and described in detail so that this method can be used in similar settings.

## Conclusion

A method was developed to generate trustworthy data about medication practice in a hospital setting by using questionnaires with predefined response options and a group of experts combining classical Delphi method elements with an item weight and performance weight decision-maker. Using a structured, transparent, predefined, and well-documented method potentially adds relevant value to research based on the widely used ISMP questionnaire. Furthermore, the method presented in this study can be reproduced like other questionnaire-based investigations that are aimed at hospital environments or similar settings. Further studies are needed to more specifically estimate value-added and establish convergence characteristics for different hospitals and subject matters.

## Data Availability Statement

The raw data of our study is in German and can only be made available upon request and with modifications that will provide data security to the participants of our work.

## Ethics Statement

Ethical approval was not provided for this study following relevant institutional and national guidelines. Since no person-sensitive data were collected, the survey was submitted and granted without an ethics committee following the current practice within our work environment at that time since no health data on a personal level were the object of our research. All participants were fully informed about the research goal and the publication. No deception was used. Consent to participate in the study was taken from all experts. Written informed consent for participation was not required under the national legislation and the institutional requirements. Data security was assured under the relevant institutional and national guidelines.

## Author Contributions

All the authors have contributed substantially to the design of the study, drafting or revising it critically, approved of the final version to be published, and agree to be accountable for the content of the study. BB-M designed the method of the decision-making process. WH ensured the appropriateness and applicability of the method. BB-M and WH wrote the article. RS contributed knowledge about questionnaire design and evaluation and assessment of medication safety. BB-M, WH, MN, SJ, and BE monitored data acquisition and analysis. SJ and MN played a leading part in data interpretation. BB-M and MN designed the flowcharts. FP collected the data for the questionnaires. BE initiated and surveyed the project.

## Funding

This study was funded by the Medical Scientific Fund of the Mayor of the City of Vienna.

## Conflict of Interest

BE is president of the Austrian Plattform Patientensicherheit/ANetPAS – Austrian Network for Patient Safety that offers access to the AMEDISS questionnaire. The remaining authors declare that the research was conducted in the absence of any commercial or financial relationships that could be construed as a potential conflict of interest.

## Publisher's Note

All claims expressed in this article are solely those of the authors and do not necessarily represent those of their affiliated organizations, or those of the publisher, the editors and the reviewers. Any product that may be evaluated in this article, or claim that may be made by its manufacturer, is not guaranteed or endorsed by the publisher.
