# Supplementary material for: Finding Consensus About the Level of Medication Safety in a Hospital Setting: Development and an Example of Application of a Modified Delphi Method
Source: Front Public Health. 2021 Sep 14;9:630398. doi: 10.3389/fpubh.2021.630398 (PMC8480327; doi:10.3389/fpubh.2021.630398)
Supplement: Supplementary file 1 [file Data_Sheet_1.docx]

Description of the flowcharts using Adonis® Software.

The elements symbolize different types of processes:

- Yellow triangle at the start = start of the process
- Dark blue rectangle = activity
- Little yellow triangles = The start and the end of subprocesses
- Red pentagons = pointers toward cross references
- Yellow diamonds = decision
- Yellow circle = end of the process

Additional explanatory comments to the flowcharts:

„Task_1: Initiating the project and getting individual answers to the questionnaire“

This flowcharts describes the process of starting a project using a questionnaire using a Delphi method/modified Delphi method.

The references to the „plausibility and completeness of data“ as quality assurance refer to form, not content. Verbatim transscription of additional remarks is ideal, but not always possible given to man-power. This can then be left out, chosing feasibilty over over-completeness.

„Task_2: Compilation of questionnaires“:

This flowchart describes the compilation of the questionnaires answered by the single experts to one answer per item. As in task 1 „plausibility and completeness of data“ refer to form, not content.

The compilation in spreadsheets is helpful to analyse the data in respect of number of answers available per item and expert opinions with same numeric value, thus making the task 3 easier.

„Task 3: Evaluation of single items oft he questionnaire in synoptic presentation“

This flowchart describes how an answer to each item is generated, taking into consideration the experts´ rating plus the evaluation of the monitor group. If this process generates a result, that is considered as „invalid“ at this stage oft he process, this will then lead to refferal of the item in question to task_4, where we show how a valid result can be generated.

„Task 4: Consequences of an invalid result“

This flowchart describes how to proceed with items of the questionnaire that have resulted in an „invalid result“ following the process described in task 3. This involves analysis and identification oft he crucial aspect of the item in question. This can lead to the insight, that the different answers within the monitor group are a result of different levels of implementation of certain structures (i.e. electronic prescribing) and strategies relevant for patient care within the faculty. The monitor can also find that the crucial aspect is only part of the job routine for a selected number of experts (i.e. handling and prescription of cytotoxic drugs) and will therefore appoint a performance weight decision maker to the respective member of the expert panel.
