# Supplementary material for: Finding Consensus About the Level of Medication Safety in a Hospital Setting: Development and an Example of Application of a Modified Delphi Method
Source: Front Public Health. 2021 Sep 14;9:630398. doi: 10.3389/fpubh.2021.630398 (PMC8480327; doi:10.3389/fpubh.2021.630398)
Supplement: Supplementary file 2 [file Data_Sheet_2.docx]

Description of the flowchart in figure 5:

This flowchart describes the respective numbers at every stage oft he process.

We started with an expert panel with 14 members, and a monitor panel with 4 members. We used one questionnaire with 276 questionnaire items, some of them with multi-part questions.

At the stage of data-acquisition this lead to 3818 ratings of the expert panel. For very specialized questions, we needid additional ratings from healthcare professionals outside the expert penal, which gave us 91 additional ratings, thus leading to 3.909 answers to the questionnaire items in total.

The monitor team screened all 3.909 of these answers and excluded 1.193 anwers that stated that the expert consulted was stating not be able to answer the question („no answer possible“).

This left 2.716 answers eligible for decision makers, and thereby for a final conclusive answer tot he item in question. Since the performance weight decision makers do not require all the answers of every expert possible, this lead to remaining 2.033 answers.

As a result we got an answer for every item, i.e. 276 items, of the questionnaire.
