# Supplementary material for: Finding Consensus About the Level of Medication Safety in a Hospital Setting: Development and an Example of Application of a Modified Delphi Method
Source: Front Public Health. 2021 Sep 14;9:630398. doi: 10.3389/fpubh.2021.630398 (PMC8480327; doi:10.3389/fpubh.2021.630398)
Supplement: Supplementary file 3 [file Data_Sheet_3.docx]

Table six:

Answer possibilites to the AMEDISS questionnaire:

- no activity to implement“
- considered, but not implemented“
- partially implemented in some or all areas“
- „completely implemented in some areas“
- „completely implemented“
- „not relevant“

Newly added in our survey: „unable to answer“
